# A Pilot Genome-Wide Association Study Identifies Potential Metabolic Pathways Involved in Tinnitus

**DOI:** 10.3389/fnins.2017.00071

**Published:** 2017-03-02

**Authors:** Annick Gilles, Guy Van Camp, Paul Van de Heyning, Erik Fransen

**Affiliations:** ^1^Department of Translational Neuroscience, Faculty of Medicine and Health Sciences, University of AntwerpWilrijk, Belgium; ^2^University Department of Otorhinolaryngology and Head and Neck Surgery, Antwerp University HospitalEdegem, Belgium; ^3^Department of Human and Social Welfare, University College GhentGhent, Belgium; ^4^Center for Medical Genetics, Antwerp University Hospital and University of AntwerpAntwerp, Belgium; ^5^StatUa Center for Statistics, University of AntwerpAntwerp, Belgium

**Keywords:** genome-wide association study, tinnitus, heritability, GWAS, phenotype, oxidative stress, serotonin, gene set enrichment analysis

## Abstract

Tinnitus, the perception of an auditory phantom sound in the form of ringing, buzzing, roaring, or hissing in the absence of an external sound source, is perceived by ~15% of the population and 2.5% experiences a severely bothersome tinnitus. The contribution of genes on the development of tinnitus is still under debate. The current manuscript reports a pilot Genome Wide Association Study (GWAS) into tinnitus, in a small cohort of 167 independent tinnitus subjects, and 749 non-tinnitus controls, who were collected as part of a cross-sectional study. After genotyping, imputation, and quality checking, the association between the tinnitus phenotype and 4,000,000 single-nucleotide polymorphisms (SNPs) was tested followed by gene set enrichment analysis. None of the SNPs reached the threshold for genome-wide significance (*p* < 5.0e–8), with the most significant SNPs, situated outside coding genes, reaching a *p*-value of 3.4e–7. By using the Genetic Analysis of Complex Traits (GACT) software, the percentage of the variance explained by all SNPs in the GWAS was estimated to be 3.2%, indicating that additive genetic effects explain only a small fraction of the tinnitus phenotype. Despite the lack of genome-wide significant SNPs, which is, at least in part, due to the limited sample size of the current study, evidence was found for a genetic involvement in tinnitus. Gene set enrichment analysis showed several metabolic pathways to be significantly enriched with SNPs having a low *p*-value in the GWAS. These pathways are involved in oxidative stress, endoplasmatic reticulum (ER) stress, and serotonin reception mediated signaling. These results are a promising basis for further research into the genetic basis of tinnitus, including GWAS with larger sample sizes and considering tinnitus subtypes for which a greater genetic contribution is more likely.

## Introduction

Tinnitus is the perception of a sound which can be perceived as hissing, ringing, buzzing, or roaring in the absence of an external sound source. It is therefore often referred to as an auditory phantom sound (Eggermont and Roberts, 2004). The prevalence of tinnitus in the adult population is ~15% (Seidman and Jacobson, 1996; Gilles et al., 2012, 2013), and in 2.5% of the population, the tinnitus causes significant burden (Axelsson and Ringdahl, 1989). The evolvement of incapacitating tinnitus may lead to social isolation, anxiety, depression, sleep disorders, and concentration issues severely affecting ones quality of life (Henry et al., 2005).

The causes of tinnitus have been extensively investigated over the years. Tinnitus appears as a symptom with increasing prevalence with age. Tinnitus appears to emerge from a complex interaction between aging, diseases, auditory malfunctions, and environmental stressors. In 50% of the cases, tinnitus is attributable to otologic malfunctions (hearing loss, noise trauma, Ménière disease, vestibular schwannoma, temporomandibular junction disorder, ototoxic medications, or substances etc.) (Gilles et al., 2014). In others, the tinnitus is a result of neurologic, metabolic, and/or psychogenic disorders (Crummer and Hassan, 2004). Environmental factors increasing the chances to develop tinnitus are recreational and work-related noise exposure (Sindhusake et al., 2003; Kim et al., 2015), smoking, sleep deprivation (Park et al., 2014; Schecklmann et al., 2015), stress (Nondahl et al., 2011), smaller households (Kim et al., 2015), and obesity (Gallus et al., 2015; Martines et al., 2015) whereas high household income and moderate alcohol consumption are considered to be inversely related to tinnitus occurrence (Nondahl et al., 2011; Gallus et al., 2015).

In contrast to the largely known environmental risk factors for tinnitus, less is known about the genes involved. Many genes have been put forward for possibly underlying tinnitus susceptibility but no consensus has been reached so far. An important complicating factor is the heterogeneity in tinnitus causes and types (Sand et al., 2010; Pawelczyk et al., 2012; Elgoyhen et al., 2014; Lopez-Escamez et al., 2016). In several monogenic disorders associated with secondary chronic tinnitus, such as neurofibromatosis type II, episodic ataxia type II, osteogenesis imperfecta type I and Fabry disease, causative genes have been identified. For an overview of monogenic diseases associated with secondary tinnitus and its associated mutated genes, we refer the reader to Sand et al. (2007). The role of genetics in the more common types of primary, chronic tinnitus in a healthy population, is under debate as data on primary chronic tinnitus heritability is very limited and family and/or twin studies are mostly lacking. The study of Kvestad et al. (2010) was the first large population-based family study to provide an estimation of the relative contribution of genetic effects on the susceptibility to tinnitus. The data were collected by use of a self-report questionnaire including questions concerning tinnitus characteristics (duration, distress, frequency) and first-degree family relationships. In total, a sample of 12,294 spouses, 27,607 parent-offspring, and 11,498 siblings was used for structural equation modeling. A heritability of 11% was obtained suggesting that the involvement of genetic factors in tinnitus is rather low (Kvestad et al., 2010).

It seems unlikely that families with hereditary tinnitus are under-reported in the literature given the large number of families reported with hereditary hearing loss. Therefore, the lack of reported families showing a Mendelian pattern of inheritance, probably indicates that primary chronic tinnitus is not a single-gene disorder but rather a complex trait, controlled by complex interactions between multiple genes and environmental risk factors. In recent years, much effort has been devoted to the genetic elucidation of the genes involved in complex traits. This showed that most complex traits and diseases are highly polygenic, involving the combined action of a multitude of genetic variants, each of which having small effect sizes. Odds ratios are typically smaller than 1.1, although variants with a larger effect size (odds ratios above 1.25) have been reported (Michailidou et al., 2015). For tinnitus, the previously estimated heritability of 11% suggests that only a small fraction of the variance is due to genetic variants. However, since the heritability represents the variance accounted for by the combined effect of several genes, it cannot be excluded that the number of involved genes is limited, and that one or more major genes account for most of the heritability. A recent study provides novel insights into the genetic contribution to tinnitus. This longitudinal study in a male twin cohort investigated the genetic contribution to tinnitus revealing a ratio of 40/60% for, respectively, genetic and environmental influences. These genetic influences for tinnitus were independent of those for hearing loss (Bogo et al., 2016). Genome-wide association studies (GWAS) are the method of choice to identify the genes involved in complex diseases. In a GWAS, genotypes from many single nucleotide polymorphisms (SNPs) throughout the entire genome (often 1 million or more) are determined and tested for association with the disease status. A strong association between a particular SNP can indicate a possible role of the variant (or the gene in which it resides) in the pathophysiology of the disease. To date, more than 2000 different disease-associated variants have been identified through GWAS analysis, providing many new insights into the pathophysiology of several diseases and traits (Welter et al., 2014).

The authors of the present manuscript previously carried out a GWAS on age-related hearing impairment (ARHI) for which a highly polygenic character of ARHI was shown, with presumably no major genes involved (Fransen et al., 2015). In the ARHI dataset, 18% of the participants reported to perceive tinnitus. The aim of the current study was to gain an insight into the genetic influence of SNPs on the tinnitus phenotype using the genome-wide SNP data from the previously collected ARHI dataset.

## Methods

### Ethics approval

The collection of the original ARHI dataset was approved by the ethics committee of the University Hospital Antwerp on October 22, 2002 (file #A02-055).

### Sample collection

The local city councils in southern Antwerp (Belgium) made population registries available by which a population-based sample could be obtained. It was requested that at least three out of the four grandparents originated from Belgium which made the population ethnically homogenous. All responding subjects underwent clinical examination, otoscopy, and completed a detailed questionnaire on medical history and exposure to environmental risk factors. A list of all questions and answers used in this study is available on request.

Strict exclusion criteria were applied to exclude persons having or having had a condition that possibly leads to hearing impairment. No phenotypic inclusion criteria were used for the sample collection. Subjects with ear diseases, possible monogenic forms of hearing impairment or other major pathologies with a possible influence on hearing, were excluded. The main goal was to study healthy subjects and therefore persons with multiple hospitalizations were excluded. The extensive, complete list of exclusion criteria was previously reported (Van Eyken et al., 2006) and is provided as Supplementary Information (Supplement 1).

Pure tone audiometry was measured in all participating subjects according to the current clinical standards (ISO 8253-1, 2010) using a two-channel Interacoustics AC-40 audiometer in a soundproof booth. Air conduction thresholds were measured at 125, 250, 500 Hz, 1, 2, 4, and 8 kHz. Bone conduction thresholds were measured within the range of 250 Hz and 4 kHz.

The phenotype of tinnitus was scored in a very basic fashion using the question “Nowadays, do you ever hear noises in your head or ear(s) (tinnitus) which usually last longer than 5 min?” No further phenotyping of tinnitus was performed as this was a retrospective study to initially identify genes involved in age-related hearing impairment (Fransen et al., 2015). Noise exposure was addressed through the question “Have you ever worked for more than a year in an environment where you had to shout to an individual standing at less than a meter from you.”

Collection of the study subjects was cross-sectional with no enrichment for any phenotype. However, the selection of the genotyped samples was based upon audiometric criteria as discussed in Fransen et al. (2015). In brief, the pure-tone thresholds from 250, 500, 1000, 2000, 4000, and 8000 Hz on age, age^2^, and age^3^ were regressed, fitting separate models for males and females. Subsequently, principal component analysis was performed on the residuals. Principal component (PC) scores for PC1, PC2, and PC3 were calculated. Since the aim was to obtain a maximal statistical power to detect genes involved in hearing loss, the phenotypically most extreme individuals for the three PC scores were selected for genotyping.

### Genotyping

DNA was extracted from blood samples using standard procedures. Genomic DNA concentrations were determined with PicoGreen (Invitrogen, Carlsbad, CA, USA). Additionally, the quality of the genomic DNA was assessed for each sample by gel electrophoresis. Five-hundred DNA samples were genotyped with the Illumina CNV370 quad chip, 1060 DNA samples using the Illumina HumanOmniExpress BeadChip (Illumina, Inc., San Diego, CA, USA).

### Imputation and filtering

Pre-imputation filtering was carried out to exclude SNPs with a minor allele frequency (MAF) below 1%, a *p*-value for Hardy-Weinberg equilibrium below 1.0e–6, a call rate below 95% across all samples and across all SNPs. Duplicate samples were identified using pi-hat analysis (pi-hat > 0.99). For each of these duplicates, the sample with the lowest genotyping call rate was removed.

Imputation was performed using the program impute2, version 2.1.2 (Howie et al., 2009) with the 1000 Genomes Phase 1 Interim panel (June 2011) as reference panel. Imputation resulted in a total of 11 626 570 SNPs. An additional post-imputation filtering was carried out using the same exclusion criteria as in the pre-imputation stage, and removing imputed SNPs with an Impute2 info metric below 0.5. Gene boundaries used for mapping SNPs onto genes are: 110 kb upstream to the most extreme gene transcript start position, and 40 kb downstream to the most extreme gene transcript end position, taking gene orientation into account.

### Association analysis

Association between the tinnitus status (affected or not affected) and the common variant genotypes (MAF > 0.01) was tested using the logistic regression option in the software package PLINK v1.07. An additive model was fitted taking the affection status as a dependent variable. This analysis tests for the main effect of a SNP on the tinnitus phenotype.

The effect of SNP genotype to noise sensitivity was tested using logistic regression by fitting a model with SNP genotype, noise exposure, and their interaction. Two significance tests were carried out on this model: first the significance on the interaction term (1df-test) was tested as well as the joint significance of the SNP genotype and the interaction term (2df-test).

### Heritability estimates

The cumulative effect of all genotyped SNPs on the phenotypic variance was estimated through variance component analysis using the genomic-relatedness-based restricted maximum-likelihood (GREML) method implemented in the Genetic Analysis of Complex Traits (GACT) software (Yang et al., 2011). All SNPs that passed the quality control were included. The Genetic Relationship Matrix was estimated correcting for incomplete Linkage Disequilibrium (LD) between genotyped and causative variants. The percentage of variance explained by the SNPs is obtained as the ratio of the genetic variance to the total variance.

The presence of genome-wide gender-specific effects was tested using a likelihood ratio test, comparing the models with and without a variance component for gene-sex interaction, as implemented in GACT.

### Gene set enrichment analysis

Gene set enrichment analysis was performed using the software package MAGENTA (Meta-Analysis Gene-set Enrichment of varianT Associations; Segre et al., 2010). This program first expresses the significance of a whole gene as a gene score, based upon the individual *p*-values of the SNPs within the gene, adjusting for confounders (gene size, number of SNPs within the gene, amount of LD, and number of recombination hotspots within the gene). Subsequently, gene scores are combined at the level of gene sets, whereby the *p*-values of a given gene set are generated by counting the fraction of the genes within that gene set that have a gene score above a predefined enrichment cut-off. As recommended by the authors, the 75th percentile of all observed gene scores was used as enrichment cut-offs to generate the *p*-values for the individual pathways. Correction for multiple testing was performed using the False Discovery Rate (FDR) method.

Analysis was performed using the *p*-values of the GWAS, using the three tests (main effects, interaction test, and joint test) as discussed earlier. SNPs were mapped to genes using Genome build36 (hg18), using gene sets from the following databases: Gene Ontology (April 2010), PANTHER (January 2010), Ingenuity (June 2008), KEGG (2010), Reactome and BioCarta.

## Results

### Sample collection

All individuals in this study were recruited through population registries in a residential suburb of Antwerp, Belgium. No enrichment for any phenotype was carried out, but strict exclusion criteria were applied to exclude individuals having or having had a disease with a possible influence on hearing. A detailed list with the exclusion criteria is provided as Supplementary Data (Supplement 1). All individuals were of Belgian ancestry. In total, 2161 individuals were randomly collected in a cross-sectional way. Of these, 1560 were selected for genotyping based upon audiometric data described in Fransen et al. (2015). Of these genotyped individuals, tinnitus information was available for 916 persons (456 females and 460 males), of which 167 (18%) were reporting episodes of tinnitus lasting longer than 5 min and the remaining 749 did not. The fraction of individuals with tinnitus was significantly different between males and females. Twenty-two percent of the males in the dataset reported tinnitus, against only 15% of the females (*p* = 0.006, chi-square test; odds ratio = 1.61 with 95% confidence interval 1.15–2.27). This difference may, at least in part, be due to a difference in noise exposure: 94 of the 167 tinnitus patients reported having worked in a noisy environment for more than a year. Only 13 of them were female, vs. 81 males (*p* < 0.001, chi-square test). Table 1 provides an overview of the characteristics of the affected and non-affected individuals in this study.

**Table 1:** **Social-demographic factors of the study population**.

	**Affected by tinnitus**	**Control**	***N***
Total population	167	749	916
Belgian ethnic origin	167 (100%)	749 (100%)	
Age range	55–65	55–65	
Mean age (st. deviation)	61.2 (3.1)	61.4 (3.1)	
Number of males (%)	100 (60%)	360 (48%)	460
Noise exposure data available	164 (98%)	733 (98%)	897
Noise exposure (%)	23 (14%)	71 (10%)	897

### Association testing

All association tests were carried out on the 916 individuals with a known tinnitus status, with the 167 tinnitus patients as cases and the 749 non-tinnitus individuals as controls.

**Figure 1:**
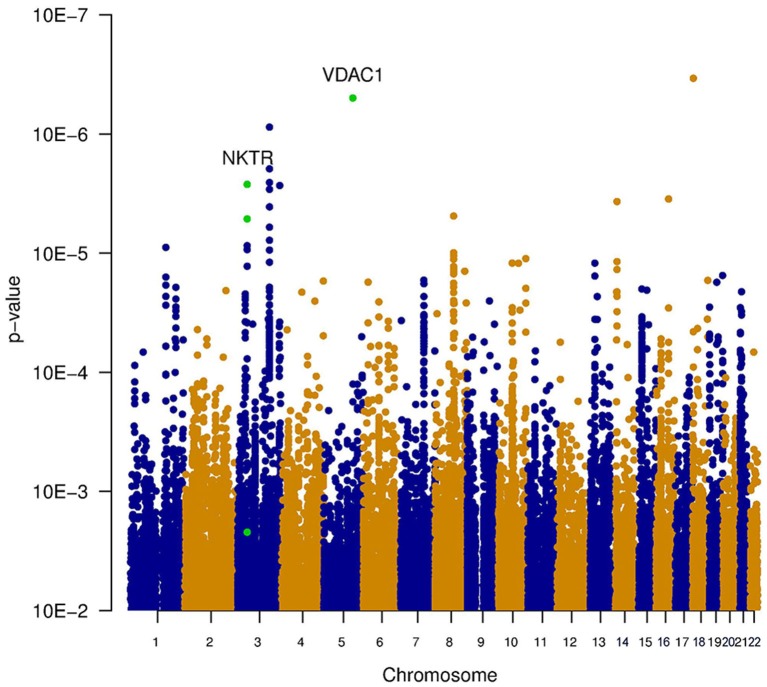
**Manhattan plot illustrating the main effect of the SNPs on the phenotype, tested via the association between tinnitus and the genotype without accounting for noise exposure**. This highlights the SNPs that lead to an increased risk for tinnitus across both noise-exposed and non-exposed individuals. None of the SNPs reached the threshold for genome-wide significance (*p* < 5e^−8^). The most significant SNPs reach a *p*-value of 3.4e^−7^. Two genes contain SNPs with a *p*-value below 1e^−5^ (VDAC1 and NKTR). These SNPs are indicated in green.

Three types of statistical tests were performed to test the effect of the SNP genotypes on tinnitus. First, the main effect of the SNPs on the phenotype was tested, via the association between tinnitus, and the genotype without accounting for noise exposure. This highlights the SNPs that lead to an increased risk for tinnitus across both noise-exposed and non-exposed individuals. Results of this analysis are shown in the table “MainEffect” (Supplement 2). None of the SNPs reached the threshold for genome-wide significance (*p* < 5.0e–8). The most significant SNPs reach a *p*-value of 3.4e–7. Two genes contain SNPs with a *p*-value below 1.0e–5 (VDAC1 and NKTR). The significance of the SNPs is graphically shown in a Manhattan plot in Figure 1, where the *p*-value is plotted vs. the chromosomal location. The SNPs within a coding gene, with a *p*-value below 1.0e–5, are shown in green.

**Figure 2:**
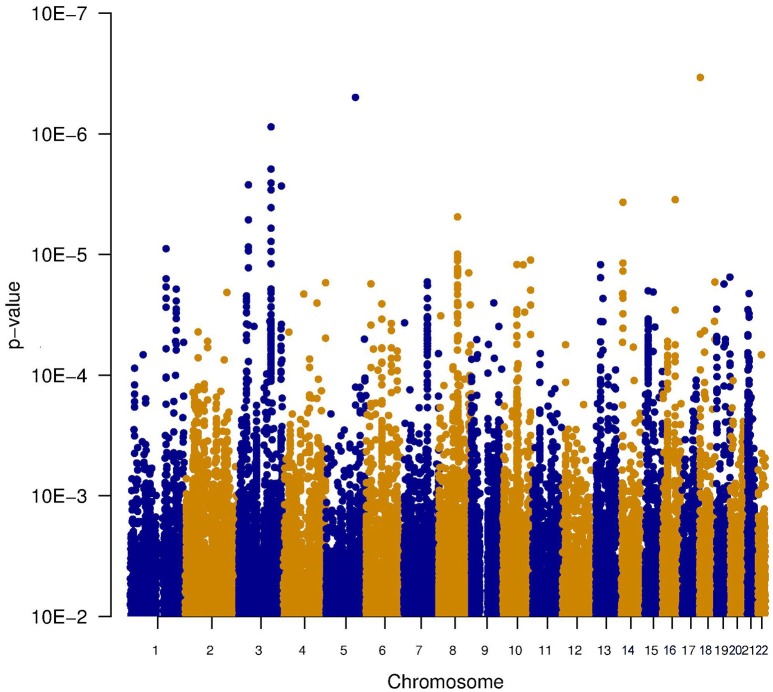
**Manhattan plot illustrating the interaction test between genotype and noise exposure**. The *p*-values test if the effect of the SNP genotype on the phenotype is altered by noise exposure. This test could highlight SNPs making an individual susceptible to tinnitus, following noise exposure.

**Figure 3:**
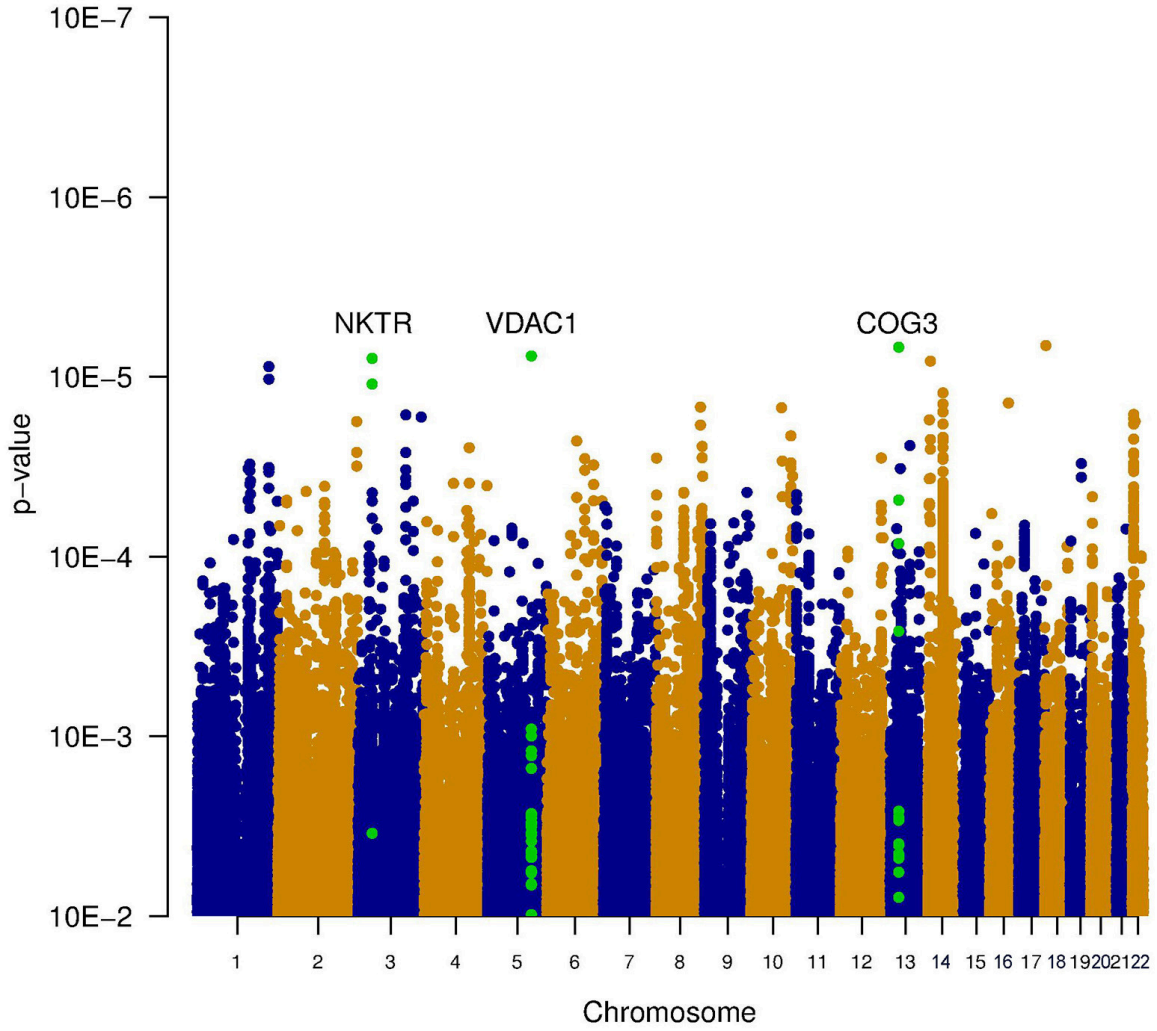
**Manhattan plot illustrating the joint test for genotype and interaction with noise**. This test combines the significance of the main effect test (Figure 1) and the interaction test (Figure 2). Two genes contain SNPs with a p-value below 1e-5 (VDAC1 and NKTR). The SNPs within these genes are shown in green.

Since it is known that noise exposure is a risk factor for tinnitus, the interaction between the SNPs and noise exposure was analyzed. This analysis would identify SNPs having a different effect on the phenotype depending on noise exposure. For instance, it could identify SNPs that render an individual susceptible to tinnitus, following noise. These SNPs would not be identified in the first analysis, as their effect on the phenotype depends on noise exposure. *P*-values for this interaction were calculated in two ways: the significance of the interaction term and the joint test for genotype and interaction with noise In general, *p*-values for the joint test are more significant than those for the interaction test, but not as significant as the *p*-values for the main effects. Genic SNPs in three genes (VDAC1, NKTR, and COG3) reach a *p*-value below 1.0e–5 for the joint test, as highlighted in the Manhattan plots (Figures 2, 3).

Tables with results are provided as Supplementary Materials (Supplement 2). These tables have been sorted by *p*-value (most significant value on top), and show the SNPs with a *p*-value below 0.0001. The far right column (“ANNOT”) shows the gene in which the SNP is located. If the SNP is further than 110 kb upstream the most extreme gene transcript start site of a coding gene, or more than 40 kb downstream to the most extreme gene transcript end position of a coding gene, this column is empty. For each of the three significance tests (main effects, interaction test and joint test), we present two tables. The first table shows all SNPs with a *p*-value below 1e–4, whereas the second one (extension “Genic”) shows the SNPs within or close to a coding gene.

### Pathway analysis

To investigate if genes, belonging to certain metabolic pathways or biological processes, were enriched in SNPs with low *p*-values, gene set enrichment analysis (GSEA) was carried out using the MAGENTA package. The analysis was performed with the results from both the main effects test, the interaction test and the joint test. Upon FDR correction for multiple testing, a total of seven pathways were significantly enriched in low *p*-values. An overview of the significant pathways is provided in Table 2. A complete overview is provided in the Supplementary Materials (Supplement 3).

**Table 2:** **Results of Gene set enrichment analysis**.

**Pathway**		**No. of genes above 75th percentile**		***p*****-value**	
	**Database**	**Expected**	**Observed no**.	**Nominal**	**FDR-corrected**
**MAIN EFFECTS**					
RAS	Panther	3	9	8.0 e–4	0.02
Vascular Smooth Muscle contraction	KEGG	26	43	1.0 e–4	0.03
**INTERACTION TEST**					
Coenzyme A biosynthesis	Panther	2	6	1.6 e–3	0.02
5HT2-type receptor mediated signaling pathway		2	6	1.4 e–3	0.03
NDK Dynamin	Biocarta	4	11	5.0 e–4	0.05
**JOINT TEST**					
NRF2-mediated oxidative stress response	Ingenuity	12	25	4.7 e–5	0.002
ER overload response	GOTERM	2	8	1.7 e–5	0.004

Using GACT software, the fraction of the phenotypic variance attributable to additive genetic effects was estimated. All genotyped SNPs passing quality control had a cumulative effect accounting for 3.2% of the phenotypic variance in the trait, with a 95% confidence interval ranging from 0 to 21%.

## Discussion and conclusions

To our knowledge, this is the first genome-wide association study on tinnitus phenotype ever performed, including 916 individuals aged between 55 and 65, consisting of 167 tinnitus cases and 749 controls. Although none of the SNPs reached the conventional threshold for genome-wide significance (*p* < 5.0e–8), and the proportion of the variance accounted for by all SNPs together is small, evidence was found for a genetic involvement in tinnitus through gene set enrichment analysis (GSEA). Throughout the three methods with which the SNPs were tested for association, seven pathways with a significant enrichment in significant SNPs emerged. On the basis of a literature search, we could not find an obvious link between tinnitus and the RAS pathway, vascular smooth muscle contraction, NDK Dynamin, and coenzyme A biosynthesis.

On the other hand, a beneficial effect of antidepressants on tinnitus has been suggested several times (Baldo et al., 2012; Beebe Palumbo et al., 2015), and this possibly involves serotonin (5HT) receptor mediated signaling pathways. Deniz et al. reported that one allelic variant of the serotonin transporter gene SLC6A4 was associated with the emotional distress associated with tinnitus (Deniz et al., 2010). Because the studies are small and often of limited quality, the results remain inconclusive at this time (Baldo et al., 2012). Moreover, since anxiety is associated with both serotonin and tinnitus (Lesch et al., 1996), it is unclear whether serotonin and tinnitus are directly associated or whether the apparent link between tinnitus and serotonin is attributable to anxiety as a confounder. The involvement of genetic variants in the serotonin mediated signaling pathways would provide a biological link between the action of antidepressants and the improvement of tinnitus (Deniz et al., 2010).

The most remarkable associations are those with endoplasmatic reticulum (ER) stress, and with the NRF2-mediated oxidative stress response. Oxidative stress plays an important role in the pathogenesis of noise-induced hearing loss and noise-induced tinnitus. Delmaghani et al. showed a direct molecular link between noise-induced hearing loss and an impaired oxidative stress defense, in a study on mice with an inactivation of the Pejvakin (*Pjvk*) gene (Delmaghani et al., 2015). They showed that in regular animals, sensory cells in the inner hair respond to noise-induced oxidative stress by upregulating *Pjvk* expression. This in turns upregulates the number of peroxysomes, which defend the cell against reactive oxygen species by restoring the normal redox balance. In mice with an inactivated *Pjvk* gene, this defense mechanism against noise-induced oxidative stress was totally absent. Following noise exposure, levels of oxidative stress were significantly increased compared to normal animals, and hearing function sharply declined. A recent study showed that tinnitus patients express significantly higher total oxidant status and oxidative index levels compared to control subjects (Koç et al., 2016) which can possibly cause dysfunctions in the microcirculation of the inner ear (Neri et al., 2002, 2006). The exact role of oxidative stress in tinnitus is still under debate. However, Honkura et al. showed that NRF2 is an undeniably crucial player in the defense mechanism against noise-induced oxidative stress (Honkura et al., 2016). A significant association between an NRF2 SNP influencing expression and susceptibility for noise-induced hearing damage was shown in noise-exposed subjects. In particular a noise notch at 4 kHz was more prevalent in subjects with a low NRF2 gene expression resulting into lower antioxidant capacity. In the current study all subjects had age-related hearing impairment, which is the most common form of sensorineural hearing loss. Oxidative stress is known to play a vital role in the development of ARHI. Along the aging process progressive decline of mitochondrial function emerges resulting into increased ROS production which in turn elicits oxidative damage and dysfunction in various tissues (Wallace, 2005). With ARHI being the most frequent cause of tinnitus development, it can be argued that besides the increase in susceptibility to hearing loss, a low NRF2 expressor allele also contributes to tinnitus development.

The endoplasmatic reticulum (ER) overload response, also referred to as ER stress or the unfolding protein response (UPR), is an evolutionary conserved response of the ER to the accumulation of unfolded proteins in the ER lumen. Although the response is primarily aimed at re-establishing the normal ER function, prolonged ER stress results in cell death through apoptosis (Xu et al., 2005). Kalinec et al. showed that the previously reported ototoxicity of N-acetyl-para-aminophenol (APAP) acts through oxidative stress and the ER overload pathway in cells derived from mouse organ of Corti (Kalinec et al., 2014). ER stress has also been linked to hearing loss in cellular and animal models for noise induced and age related hearing loss (Van Rossom et al., 2015; Hu et al., 2016; Xue et al., 2016). ER stress was suggested to be the earliest molecular event leading to hearing loss (Hu et al., 2016), and progression could be delayed with an ER stress inhibitor.

The identification of pathways involved in tinnitus is a useful first step into a better understanding of the pathophysiology and the identification of possible drug targets. A further step would be the identification of individual SNPs associated with tinnitus, which would deepen our understanding how the symptom arises on a molecular basis. A potential follow-up study could therefore focus on genotyping SNPs in genes belonging to the pathways identified in the current study.

It is not surprising that the current study has not been able to find individual SNPs associated with tinnitus. First of all, this sample set was initially not optimized to study the genetic causes of tinnitus. It was collected as a cross-sectional study to investigate age-related hearing impairment, and no selective enrichment for tinnitus patients was carried out. The observed prevalence of 18% is in line with previously reported epidemiological studies (Axelsson and Ringdahl, 1989; Gilles et al., 2013). There was no matching for other tinnitus risk factors, although noise exposure was accounted for in the analysis stage. Hence, the current dataset had limited power to detect individual SNPs associated with tinnitus. A sensitivity analysis using the Genetic Power calculator (Purcell et al., 2003) showed that the current dataset holds 80% power to detect an allele with a genotype relative risk of 2, under an additive model, at a genome-wide significance level (*p* < 5e–8). Since no genome-wide significant SNPs were found in the current GWAS, it can be concluded that presumably, no major genes with a genotype relative risk of 2 or larger in tinnitus exist.

The finding of several pathways significantly enriched in significant SNPs, seems in contradiction with the lack of SNPs reaching genome-wide significance and the low heritability. The genetic signal, picked up using the GSEA, must therefore reside in the SNPs reaching a nominal, but not a genome-wide, significance level. For instance, for both the main effect test and the joint test, there are about 700 SNPs (both genic and non-genic) reaching a *p*-value below 1.0e–4. Due to the multitude of hypotheses tested in a GWAS, it is quite likely that a large part of these association signals represent false positives, but a fraction of the SNPs with low *p*-values will represent a genuine association signal. This is in line with previous studies into, amongst others, psychiatric diseases and age-related hearing loss, where the SNPs with a low *p*-value not reaching genome-wide significance, still hold substantial genetic information on the phenotype (International Schizophrenia Consortium et al., 2009; Fransen et al., 2015).

A very low heritability estimate was obtained through variance components analysis as implemented in GACT. This showed that the cumulative effect of all SNPs in the GWAS on the phenotype only explains 3.2% of the phenotypic variance in tinnitus. Several explanations are possible to reconcile this weak effect of genes with the presence of genetic signals through the GSEA. A first explanation is the inaccuracy of the current estimate due to the small sample size. The standard error of the estimate was 9.2%, which leads to a confidence interval for the heritability ranging from 0 to 21%. The previously reported heritability of 11% by Kvestad et al. (2010), falls within this 95% confidence interval. Moreover, the GCTA-GREML method implemented in GCTA only accounts for the additive genetic effects of the SNPs in the GWAS. The actual genetic effect may be larger due to, amongst other, rare SNPs that are not analyzed in a GWAS or SNP-SNP interactions. The combination of this very weak effect of all individual SNPs combined, with the significant results of the GSEA, seems to indicate that only a very limited number of SNPs are involved in tinnitus, but these SNPs seem to be confined in a small number of disease-associated pathways.

Although GWASs have been very successful in the identification of disease genes, the technique has its limitations. Most SNP variants genotyped in the current GWAS are common, typically with a minor allele frequency of at least 5%. The effects of low-frequency and rare SNPs are not captured. Novel techniques, such as next-generation sequencing, now allow to more thoroughly study the role of rare variants in complex diseases, and traits (Rivas et al., 2011; Gudmundsson et al., 2012). Moreover, intense research is being carried out on the role of non-coding variation in the genome. Since most SNPs identified in GWAS are located outside coding genes, and probably reside within regulatory regions, subtle alterations in gene expression level may be a major player in complex traits and diseases. However, there are very few non-coding SNPs for which the exact role in gene expression has already been elucidated.

The ideal study design, for a genetic study on tinnitus, would be a case-control dataset with an equal number of tinnitus cases and matched controls taking into account potential confounders such as anxiety, age, hearing loss, occupational/recreational noise exposure, and distress. Since most genetic effect sizes for complex traits are small, with the variance explained by a single SNP in the order of 0.1% of the phenotypic variance and genotype relative risks around 1.1, detecting such risk alleles is only feasible using massive sample sizes of 10,000 individuals, collected through large, international consortia. In addition, these studies should more carefully study the tinnitus phenotype and take into account the clinical heterogeneity of the trait. In the current study, the anamnesis was based upon one simple question in a questionnaire, without any distinction between the various subgroups of tinnitus. Tinnitus phenotype has proven to be very heterogeneous and, as such, the tinnitus phenotype in the present study was inevitably heterogeneous as well. Further improvement can be reached by construction of a standardized operation protocol to collect and phenotype tinnitus patients and controls. Limiting the tinnitus patients to certain subtypes, excluding for example somatic tinnitus for which a non-genetic etiology is likely (Lopez-Escamez et al., 2016), would increase the power of genetic studies into tinnitus.

So far no large GWAS into tinnitus has been carried out. The lack of large international studies into tinnitus genetics is possibly attributable to the general assumption that tinnitus is a symptom rather than a trait with few genetic factors involved. Interestingly, a recent longitudinal twin study (Bogo et al., 2016) estimated the proportion of additive genetic factors in tinnitus at 40%, which is substantially higher than the 11% previously estimated by Kvestad (Kvestad et al., 2010). Together with the results from the current study, the idea seems to emerge that the role of genetic factors in tinnitus is larger than initially assumed, and that the identification of SNPs involved in tinnitus is feasible if sufficiently large sample sets of well-characterized tinnitus and control subjects are collected.

## Author contributions

AG wrote the manuscript, co-operated in the statistical analyses, and is the corresponding author. GV and PV were involved in the design of the study, the collecting of the original data, and reviewed the draft manuscript. EF performed statistical analyses and co-operated in writing the manuscript.

### Conflict of interest statement

The authors declare that the research was conducted in the absence of any commercial or financial relationships that could be construed as a potential conflict of interest. The reviewer CC declared a past co-authorship with one of the authors PV to the handling Editor, who ensured that the process met the standards of a fair and objective review.

## Supplementary material

### Public data access

Data from this study are publicly available through the following link: http://bit.ly/2jYmRii.
