# Supplementary material for: A Pilot Genome-Wide Association Study Identifies Potential Metabolic Pathways Involved in Tinnitus
Source: Front Neurosci. 2017 Mar 2;11:71. doi: 10.3389/fnins.2017.00071 (PMC5332393; doi:10.3389/fnins.2017.00071)
Supplement: Supplementary file 1 [file DataSheet1.DOCX]

**Supplementary material: Inclusion and exclusion criteria**

***1. Syndromic hearing impairment***

Subjects who are suspected to have a syndrome associated with hearing loss were excluded. Special attention was therefore given to:

a. The auricle: is there microtia or a bat-ear?

b. The pre-auricular area: are there appendages or pits, or a pre-auricular sinus?

c. The ear canal: is it normally formed?

d. The tympanic membrane and ossicles: are there malformations?

e. Other syndromic features:

- Facial dysmorphia
- Abnormal position or shape of the eyes
- Schisis
- Pigment abnormalities
- Low hairline
- Goiter

Subjects with any of these characteristics or symptoms are not included in the study.

***2. Middle ear pathology***

These subjects have to be excluded as well. Very important for the diagnosis of middle ear disease is the otoscopic examination. Therefore we propose a classification of the tympanic membrane appearance as follows ([Browning and Gatehouse, 1992](#_ENREF_1)).

a. Normal tympanic membrane: an intact tympanic membrane with no areas of substantial scarring, thinning, retraction, tympanosclerosis or localized chalk patches

b. Abnormal tympanic membrane: an intact tympanic membrane but substantial scarring, thinning, retraction, tympanosclerosis or chalk patches

c. Acute otitis media: acute otitis media or otitis media with effusion

d. Inactive chronic otitis media: the presence of a tympanic membrane perforation without evidence of inflammation in the form of middle ear mucosal oedema or pooling of secretions

e. Active chronic otitis media: the presence of a perforated tympanic membrane with active inflammation either due to mucosal disease or a cholesteatoma

Patients with otological disease in either ear are excluded from the study.

***3. Audiological and clinical otological exclusion criteria***

Patients with ear diseases other than presbycusis that affect hearing thresholds have to be excluded from the study. Subject exclusion can be based on audiological, tympanometric or otological criteria. Furthermore, some sensorineural hearing losses have to be excluded as well. To make our population under study as homogeneous as possible, we also want to exclude all subjects with a major pathology that might influence hearing. In addition, people with a cochlear implant should not be included.

***3.1 Clinical criteria***

A subject with one or both tympanic membrane appearances scored as acute otitis media, inactive chronic otitis media or active chronic otitis media (see also guidelines for clinical examination) are excluded from the study, just like subjects with syndromic features or congenital otological abnormalities.

***3.2 Sensorineural hearing impairment***

Subjects with one of the following diseases are excluded from the study.

a. Menière’s disease

b. VIII nerve tumor

c. Ramsay Hunt Syndrome

d. Post-meningitis

e. All sudden losses

f. Other genetic hearing losses

***3.3 Audiological criteria***

a. Conductive hearing loss
If there is an air-bone gap averaged over 0.5, 1 and 2 kHz of ≥ 15dB in one or both ears: exclusion of this subject from the study.

b. Asymmetrical hearing loss
If there is a difference between left and right ear air conduction thresholds of ≥20dB for at least 2 frequencies out of 0,5; 1; and 2 kHz

Both reasons lead to exclusion of this subject from the study.

**4. Exclusion criteria on basis of general pathology**

***4.1 List of included and excluded pathologies***

This list of what to include and what to exclude is a guideline but will certainly not be exhaustive. ***IN*** means include in the study, ***EX*** means exclude from the study.

1. cardiovascular disease
   1. cardiac disease
      1. coronary disease ***IN***
         1. angina pectoris
         2. infarction
         3. percutaneous transluminal coronnary arteriography
      2. congenital heart abnormalities ***IN***
      3. cardiac valve pathology ***IN***
      4. arrhythmia ***EX***
      5. cardiac failure ***EX***
      6. cardiac transplantation ***EX***
   2. pathology of the carotid arteries ***IN***
      1. stroke TIA/CVA ***EX***
      2. carotid surgery ***EX***
   3. pathology of the femoral/popliteal arteries ***IN***
   4. pathology of the abdominal arteries ***IN***
   5. pathology of the renal arteries
      1. renal hypertension ***IN***
   6. hypertension
      1. primary ***IN***
      2. adrenal hyperplasia ***IN***
   7. hypercholesterolemia *(****IN****, but carefully write down if high or low)*
2. hormonal/metabolic disease
   1. hormonal
      1. diabetes *(****IN*** *but carefully write down duration and therapy)*
         1. type I
         2. type II
      2. thyroid disease *(****IN****,but carefully write down duration and therapy)*
         1. hypothyroidy
         2. hyperthyroidy
      3. hyperparathyroidism ***IN***
      4. other ***EX***
         1. Cushing’s disease
         2. Addisson’s disease
         3. acromegaly
         4. hyperprolactinemia
         5. diabetes insipidus
         6. pheochromocytoma
         7. …
   2. metabolic
      1. osteoporosis ***IN***
      2. renal disease ***EX***
         1. chronic renal insufficiency
         2. hemodialysis
         3. transplantation
      3. liver disease ***EX***
         1. haemochromatosis
         2. cirrosis
         3. chronic liver failure
         4. liver transplantation
         5. Gilbert’s disease ***IN***
3. Autoimmune diseases all ***EX***
   1. Rheumatoid arthritis ***EX***
   2. Lupus erythematosus ***EX***
   3. Inflammatory bowel disease ***EX***
   4. Ankylosing spondilitis ***EX***
   5. Temporal arteritis ***EX***
   6. Gout ***EX***
   7. Cogan’s disease ***EX***
   8. Bechet’s disease ***EX***
   9. Wegener’s granulomatosis ***EX***
   10. IgA nepropathy ***EX***
   11. Takayasu’s disease ***EX***
   12. Polyarteritis nodosa ***EX***
   13. Scleroderma ***EX***
   14. Dermatomyositis ***EX***
   15. Sjögrens disease ***EX***
   16. Other ***EX***
4. Neoplasms
   1. In the ear ***EX***
   2. With therapeutic radiotherapy onto the region of the ear ***EX***
   3. With therapeutic chemotherapy ***EX***
   4. Haematological neoplasms (leukemia, lymfoma, Hodgkin’s disease, polycythemia vera, m.waldenström, amyloïdose…) ***EX***
   5. Brain tumors ***EX***
   6. Metastatic neoplasms ***EX***
   7. Other: local neoplasms ***IN***

*General: no head and neck cancers. No chemotherapy, no local (head) radiotherapy*

1. Neurological disease
   1. (Alzheimer’s) dementia ***EX***
   2. Parkinsons disease ***EX***
   3. Multiple sclerosis ***EX***
   4. Epilepsy ***IN***
   5. Migraine ***IN***
   6. Other ***EX***

*This implies all neurological diseases EX, except epilepsy and migraine*

1. Pyschiatric disease
   1. Severe mental illnesses for which hospitalization has been necessary ***EX***
   2. Other ***IN***
2. Pulmonary disease
   1. COPD/astma/emphysema ***IN***
   2. Pneumoconiose – asbestose - silicose ***IN***
   3. Idiopathic pulmonary fibrosis ***EX***
   4. Cystic fibrosis ***EX***
   5. Sarcoidosis ***EX***
3. Haematological disease
   1. Sickle cell anemia ***EX***
   2. Haemophilia ***EX***
   3. Von Willebrand’s disease ***EX***
   4. Anemia ***IN***
   5. Any haematological disease for which the subject is being treated ***EX***
4. Dermatological, ophtalmological, gynecological disease; diseases of the stomach or bowels ***IN***
5. Infectious diseases
   1. AIDS ***EX***
   2. Syphilis ***EX***
   3. Lyme disease ***EX***
   4. Hepatitis B or C ***EX***
   5. Tuberculosis ***EX***
   6. Meningitis ***EX***
   7. Herpes Zoster ***EX***
   8. Other *(include but specify)*
6. Other subjects to ***ex***clude
   1. All congenital syndromes (Down’s syndrome etc.)
   2. All rare diseases that are severe enough to cause significant handicap according to the subject

Browning, G.G., and Gatehouse, S. (1992). The prevalence of middle ear disease in the adult British population. *Clin Otolaryngol Allied Sci* 17**,** 317-321.
